# Different Factors Associated with CTX-M-Producing ST131 and Non-ST131 *Escherichia coli* Clinical Isolates

**DOI:** 10.1371/journal.pone.0072191

**Published:** 2013-09-04

**Authors:** Marie-Hélène Nicolas-Chanoine, Jérôme Robert, Marie Vigan, Cédric Laouénan, Sylvain Brisse, France Mentré, Vincent Jarlier, the Coli β study group

**Affiliations:** 1 Service de Microbiologie, Hôpital Beaujon AP-HP, Clichy, France; 2 Faculté de Médecine D. Diderot, Paris, France; 3 Institut National de la Santé et de la Recherche Médicale, U773, Centre de Recherche Biomédicale Bichat-Beaujon (CRB3), Université Paris Diderot, Paris, France; 4 Bactériologie-Hygiène, EA 1541/ER 5, Université Pierre et Marie Curie-Paris 6, Site Pitié, Paris, France; 5 Hôpitaux universitaires Pitié Salpêtrière-Charles Foix, AP-HP, Paris, France; 6 UMR-S 738 INSERM - Université Paris Diderot, Sorbonne Paris Cité, Paris, France; 7 Service de Biostatistique, Hôpital Bichat AP-HP, Paris, France; 8 Institut Pasteur, Plateforme Génotypage des Pathogènes et Santé Publique, Paris, France; Institut National de la Recherche Agronomique, France

## Abstract

**Objectives:**

To determine factors associated with CTX-M-producing ST131 *Escherichia coli* which is the worldwide predominant lineage among CTX-M-producing *E. coli* isolates.

**Methods:**

Consecutive inpatients with a clinical sample positive for CTX-M-producing *E. coli* and considered as cases in a previous 8-month (2008–2009) case-control study performed in ten university hospitals in the Paris area were included in the present sub-population study. Patients with a CTX-M-producing ST131 *E. coli* clinical isolate were compared with those with a CTX-M-producing non-ST131 *E. coli* clinical isolate with regard to 66 variables. Variables were first compared using univariate logistic regression, then a multivariate analysis using a backward selection with variables with p-value <0.1 in univariate analysis was carried out.

**Results:**

Fifty-five patients with a CTX-M-producing ST131 *E. coli* clinical isolate were compared to 97 patients with a CTX-producing non-ST131 *E. coli* clinical isolate. Multivariate analysis showed that only previous residence in long term care facilities (OR = 4.4; 95% CI = 1.3–14.7) was positively associated with a CTX-M-producing ST131 *E. coli* isolate. However, it also showed that regular consumption of poultry products (OR = 0.2; 95% CI = 0.1–0.6), having had at least one device in the preceding 6 months (OR = 0.3; 95% CI = 0.1–0.7) and stay in ICU (OR = 0.2; 95% CI = 0.05–0.8) were negatively associated with isolation of CTX-M-producing ST131 *E. coli* from clinical samples.

**Conclusions:**

This study provides more insight into the epidemiological features of ST131 and non-ST131 *E. coli* producing CTX-M enzymes. It shows, for the first time, that isolation of CTX-M-producing ST131 *E. coli* from clinical samples is not linked to consumption of various foods and confirms that residence in long term care facilities is a predictor of these isolates.

## Introduction

The polyclonal structure of *Escherichia coli* from clinical and commensal human isolates, and from environmental isolates has clearly been shown by studies recently carried out in the Netherlands (clinical and commensal human isolates and chicken meat isolates), England (clinical isolates) and France (clinical and commensal isolates) on the basis of sequence types (ST) [1]–[5]. However, some *E. coli* lineages were identified as predominant in the five above cited studies independent of the source of the isolate or the production of extended-spectrum β-lactamase (ESBL). In particular, *E. coli* ST131 was predominant among the clinical and commensal human isolates, producers of ESBL or not. In contrast, it was not identified in ESBL-producing *E. coli* isolated from chicken meat in the Netherlands. The absence of clone ST131 has also been confirmed recently in Spain among the *E. coli* isolates contaminating raw chicken meat [6] although another previous Spanish study had found that 7% of retail chicken samples were contaminated by *E. coli* ST131 [7]. Vincent *et al*. also had identified *E. coli* ST131 from retail chicken samples in Canada but at a significant lower prevalence (0.4%) than in Spain [8]. In contrast, isolates of ST10, comprising ESBL and non-ESBL producers, were frequent both among the clinical and commensal human isolates as well as among the meat isolates [1], [2], [4], [5], [8]. As shown by the Dutch, Canadian and French studies, the recognized avian pathogenic *E. coli* ST117 was another predominant lineage among the clinical and meat isolates [1], [5], [9]. On the other hand, although CTX-M-15 was shown to be the predominant CTX-M enzyme (46%) among the French clinical isolates, it should be stressed that CTX-M-1 was the only ESBL found in the ST117 clinical isolates in France and was the predominant ESBL found among the ST117 meat isolates in the Netherlands [1], [5].

These reports suggest that epidemiological differences exist between CTX-M-producing strains of ST131 and non-ST131 clones. Therefore, we sought to analyse characteristics associated with CTX-M-producing *E. coli* ST131 isolated from clinical samples by performing a sub-population analysis of data collected during a case-control study carried out from November 2008 to June 2009 to determine factors independently associated with a clinical sample positive for a CTX-M-producing *E. coli* isolate in ten hospitals of the Paris area [10]. The analysis of the population structure of CTX-M-producing *E. coli* and non-ESBL-producing *E. coli* isolates which was performed in addition to the case-control study, was also used as a basis for the present study [1].

## Materials and Methods

### Ethics Statement

Written informed consent was obtained from all adult participants and from parents for child participants. The study and the consent procedure were approved by the Ethics Committee of the Groupe Hospitalier Universitaire Nord (Institutional review board N°IRB00006477).

### Study Design and Participants

All consecutive inpatients with a clinical sample positive for CTX-M-producing *E. coli* and considered as cases in a previous 8-month (2008–2009) case-control study performed in ten university hospitals in the Paris area were included in the present sub-population study [10]. Patients with a clinical sample positive for CTX-M-producing ST131 *E. coli* (n = 55) were compared with those with a clinical sample positive for CTX-M-producing non-ST131 *E. coli* (n = 97) with regard to 66 characteristics collected during the case-control study, including basic demographic data, patient’s lifestyle (housing, travel abroad, diet, pet, sport practice…), medical history (hospitalisation and invasive devices in the preceding six months, antibiotic in the preceding month, co-morbidity…) and data on the current hospitalisation (hospitalisation wards**,** invasive devices, antibiotic regimens …). The 97 non-ST131 *E. coli* isolates displayed 51 ST types of which 38 were displayed by a single isolate and 13 by several isolates: 14 isolates for ST10, 7 for ST167 and ST648, 4 for ST88 and ST410, 3 for ST38, ST93, ST117, ST354, ST405, ST617 and ST1284 and 2 for ST44 [1].

### Statistical Analysis

Variables were first compared using univariate logistic regression and odds ratio (OR) and 95% confidence interval (CI) were estimated. We next used a multivariate analysis using a backward selection with variables with p-value <0.1 in univariate analysis. P-values were assessed at the 0.05 level. All statistical analyses were performed with SAS software, version 9.3 (SAS Institute, Cary, North Carolina).

## Results

A total of 55 patients with a CTX-M-producing ST131 *E. coli* clinical isolate were compared to 97 patients with a CTX-M-producing non-ST131 *E. coli* clinical isolate with regard to the 66 variables studied (Tables 1, 2 and 3). In univariate analysis, patients harbouring *E. coli* ST131 were more likely than those harbouring non-ST131 *E. coli* to be aged >65 years (OR = 2.2; 95% CI = 1.1–4.3) and >80 years (OR = 3.8; 95% CI = 1.8–7.7) (Table 1). Among factors focusing on patient’s lifestyle (Table 1), living in collective housing (OR = 3.9; 95% CI = 1.6–9.3) and being functionally dependent before hospitalisation (OR = 4.3; 95% CI = 2.1–8.8) were significantly associated with a ST131 *E. coli* clinical isolate. On the opposite, consumption of poultry at least twice a week was inversely associated with a ST131 *E. coli* clinical isolate (OR = 0.3; 95% CI = 0.1–0.7) (Table 1). Patients with a ST131 *E. coli* clinical isolate were more likely than others to have been in long term care facilities (LTCF) between admission and study inclusion (OR = 2.8; 95% CI = 1.2–6.3), and to have a urinary tract infection during the current hospitalisation (OR = 2.2; 95% CI = 1.0–4.6) (Table 3). On the opposite, patients with a ST131 *E. coli* clinical isolate were less likely to have surgery in the last month (OR = 0.4; 95% CI = 0.2–0.9) (Table 2), to have been in intensive care unit (ICU) (OR = 0.3; 95% IC = 0.1–0.9), and to have invasive devices within the week prior inclusion (OR = 0.2; 95% CI = 0.1–0.5), notably a urinary catheter (OR = 0.3; 95% CI = 0.1–0.6), and intravascular devices (OR = 0.2; 95% CI = 0.1–0.5) (Table 3).

**Table 1:** Univariate and multivariate analyses of demographic and lifestyle factors associated with a CTX-M-producing ST131 or non-ST131 *E. coli* clinical isolate.

	Univariate analysis				Multivariate analysis		
Characteristic	ST131 (n = 55)	Non-ST131 (n = 97)	Odds ratio	P value	Odds ratio	P value	
	No. (%)	No. (%)	(95% CI)		(95% CI)		
*Demographic data*							
Age (mean ± SD) in years	70.2±25.8	60.5±24.0	1.0 (1.0–1.0)	0.02			
Age <15 years	3 (5.4)	6 (6.2)	0.9 (0.2–3.6)	0.8			
Age ≥ 65 years	37 (67.3)	47 (48.5)	2.2 (1.1–4.3)	0.03			
Age ≥80 years	28 (50.9)	21 (21.7)	3.8 (1.8–7.7)	0.0003			
Female	41 (74.6)	58 (59.8)	2.0 (1.0–4.1)	0.07			
Country of birth outside of Europe	15 (27.3)	36 (37.1)	0.6 (0.3–1.3)	0.2			
Living in a country outside of Europe	1 (1.8)	10 (10.3)	0.2 (0.02–1.3)	0.09			
*Lifestyle*							
Collective housing	17 (30.9)	10 (10.3)	3.9 (1.6–9.3)	0.002			
Individual housing (>2 household members)	11 (20.0)	33 (34.0)	0.5 (0.2–1.1)	0.07			
Live alone	13 (23.6)	21 (21.7)	1.1 (0.5–2.5)	0.8			
Functionally dependent before hospitalisation	29 (52.7)	20 (20.6)	4.3 (2.1–8.8)	<0.0001			
Patients not working	43 (78.2)	64 (66.0)	1.8 (0.9–4.0)	0.12			
Retired patients	37 (67.3)	51 (52.6)	1.9 (0.9–3.7)	0.08			
Consumption of:							
- ≥7 raw vegetables/week	26 (68.4)	59 (69.4)	1.0 (0.4–2.2)	0.9			
- poultry ≥ twice a week	15 (39.5)	56 (66.7)	0.3 (0.1–0.7)	0.006	0.2 (0.1–0.6)	0.002	
- beef ≥ twice a week	21 (55.3)	57 (67.9)	0.6 (0.3–1.3)	0.2			
Consumption of raw meat	9 (16.4)	28 (28.9)	0.5 (0.2–1.1)	0.09			
Community meal	29 (52.7)	52 (53.6)	1.0 (0.5–1.9)	0.9			
Practice of a sport	3 (5.5)	8 (8.3)	0.6 (0.2–2.5)	0.5			
Pets or livestock	5 (9.1)	14 (14.4)	0.6 (0.2–1.7)	0.3			
Travel abroad in the preceding 6 months	3 (5.5)	14 (14.4)	0.3 (0.09–1.2)	0.1			

**Table 2:** Univariate and multivariate analyses of medical history-related factors associated with a CTX-M-producing ST131 or non-ST131 *E. coli* clinical isolate.

	Univariate analysis				Multivariate analysis	
Characteristic	ST131 (n = 55)	Non-ST131 (n = 97)	Odds ratio	P value	Odds ratio	P value
	No. (%)	No. (%)	(95% CI)		(95% CI)	
In the preceding 6 months						
- hospitalised	34 (61.8)	63 (65.0)	0.9 (0.4–1.7)	0.7		
- hospitalised ≥10 days	19 (34.6)	42 (43.3)	0.7 (0.3–1.4)	0.3		
- hospitalised <10 days	15 (27.3)	21 (21.7)	1.4 (0.6–2.9)	0.4		
- hospitalised outside of France	1 (1.8)	7 (7.2)	0.2 (0.03–2.0)	0.2		
- at least one invasive device	30 (54.6)	66 (68.0)	0.6 (0.3–1.1)	0.1	0.3 (0.1–0.7)	0.01
• urine drainage	16 (29.6)	31 (32.3)	0.9 (0.4–1.8)	0.7		
• mechanical ventilation	3 (5.6)	10 (10.4)	0.5 (0.1–1.9)	0.3		
• intravascular devices	29 (53.7)	62 (64.6)	0.6 (0.3–1.3)	0.2		
• colonoscopy, endoscopy	9 (17.3)	28 (30.4)	0.5 (0.2–1.1)	0.09		
Surgery during the last month	10 (18.2)	34 (35.4)	0.4 (0.2–0.9)	0.03		
Prothesis within the last year	2 (3.7)	8 (8.3)	0.4 (0.09–2.1)	0.3		
Antibiotic in the month preceding hospitalisation	16 (29.1)	37 (38.1)	0.7 (0.3–1.4)	0.3		
- cotrimoxazole	2 (3.6)	8 (8.3)	0.4 (0.09–2.1)	0.3		
- fluoroquinolones	4 (7.3)	7 (7.2)	1.0 (0.3–3.6)	1.0		
- extended spectrum cephalosporins	4 (7.3)	7 (7.2)	1.0 (0.3–3.6)	1.0		
- penicillins	6 (10.9)	11 (11.3)	1.0 (0.3–2.7)	0.9		
- ≥5 days	9 (16.4)	22 (22.7)	0.7 (0.3–1.6)	0.4		
Nursing or physiotherapy before hospitalisation	9 (16.4)	17 (17.5)	0.9 (0.4–2.2)	0.9		
At least one co-morbidity	33 (60.0)	57 (58.8)	1.1 (0.5–2.1)	0.9		
- recurrent urinary tract or chronic skin infections	18 (32.7)	21 (21.7)	1.8 (0.8–3.7)	0.1		
- obstructive bronchial pulmonary disease	2 (3.6)	5 (5.2)	0.7 (0.1–3.7)	0.7		
- cancer	10 (18.2)	27 (27.8)	0.6 (0.3–1.3)	0.2		
- diabetes	12 (21.8)	22 (22.7)	0.9 (0.4–2.1)	0.9		

**Table 3:** Univariate and multivariate analyses of current hospitalisation-related factors associated with a CTX-M-producing ST131 or non-ST131 *E. coli* clinical isolate.

	Univariate analysis				Multivariate analysis	
Characteristic	ST131 (n = 55)	Non-ST131 (n = 97)	Odds ratio	P value	Odds ratio	P value
	No. (%)	No. (%)	(95% CI)		(95% CI)	
Transferred from another hospital	32 (25.5)	18 (18.6)	1.5 (0.7–3.3)	0.3		
Mc Cabe score 2	11 (21.6)	23 (27.1)	0.7 (0.3–1.7)	0.5		
Immunocompromised	15 (27.3)	36 (37.1)	0.6 (0.3–1.3)	0.2		
Between admission and inclusion						
- Ward						
• ICU	4 (7.3)	27 (27.8)	0.3 (0.1–0.9)		0.2 (0.05–0.8)	0.02
• LTCF	21 (38.2)	14 (14.4)	2.8 (1.2–6.3)	0.0009*	4.4 (1.3–14.7)	0.02
• Others	30 (54.5)	56 (57.8)	1		1	
- Invasive device during the last week	33 (60.0)	84 (86.6)	0.2 (0.1–0.5)	0.0003		
• urine drainage	11 (20.0)	45 (46.4)	0.3 (0.1–0.6)	0.002		
• mechanical ventilation	5 (10.6)	19 (20.0)	0.5 (0.2–1.4)	0.2		
• intravascular devices	31 (56.4)	81 (84.4)	0.2 (0.1–0.5)	0.0003		
- Antibiotic receipt	24 (43.6)	57 (58.8)	0.5 (0.3–1.1)	0.07		
• cotrimoxazole	3 (5.5)	6 (6.2)	0.9 (0.2–3.6)	0.9		
• fluoroquinolones	7 (12.7)	10 (10.3)	1.3 (0.5–3.5)	0.7		
• penicillins	10 (18.2)	26 (26.8)	0.6 (0.3–1.4)	0.2		
• extended spectrum cephalosporins	6 (10.9)	12 (12.4)	0.9 (0.3–2.5)	0.8		
• aminoglycosides	2 (3.6)	11 (11.3)	0.3 (0.06–1.4)	0.1		
• carbapenems	1 (1.8)	8 (8.3)	0.2 (0.03–1.7)	0.14		
• ≥5 days	13 (23.6)	35 (36.1)	0.5 (0.3–1.2)	0.1		
Specimen and infection data						
- specimen sampled after 48 h of hospitalisation	35 (63.6)	51 (52.6)	1.6 (0.8–3.1)	0.2		
- specimen sampled after >10 days of hospitalisation	21 (38.2)	35 (36.1)	1.1 (0.6–2.2)	0.8		
- urine sample	39 (70.9)	58 (59.8)	1.6 (0.8–3.3)	0.2		
- urinary tract infection	42 (76.4)	58 (59.8)	2.2 (1.0–4.6)	0.04		

ICU; intensive care unit, LCTF; long term care facility,

* P value resulting from the analysis of the variable “ward” classified into 3 categories, *ie* ICU, LCTF and others.

In multivariate analysis, only previous residence in LTCF (OR = 4.4; 95% CI = 1.3–14.7) remained positively associated with *E. coli* ST131 (Table 3). However, consumption of poultry at least twice a week (OR = 0.2; 95% CI = 0.1–0.6) (Table 1), having had at least one device in the preceding 6 months (OR = 0.3; 95% CI = 0.1–0.7) (Table 2), and hospitalisation in ICU (OR = 0.2; 95% CI = 0.05–0.8) (Table 3) were, independently, inversely associated with isolation of *E. coli* ST131 from clinical samples.

## Discussion

*E. coli* ST131 has been shown to be a worldwide predominant clone among extra-intestinal pathogenic isolates but also among the human commensal flora [2], [4], [10]–[12]. Interestingly, it was found to be almost the only lineage among clinical isolates of group B2 *E. coli* that produced CTX-M enzymes [1]. It displayed a higher ability to colonize the digestive tract and a lower level of virulence in various animal models in comparison with reference group B2 urinary pathogenic *E. coli* strains (CFT053, J536 and HT7) [13]–[15]. Therefore, better knowing the epidemiology of clone ST131, which appears to be a very peculiar group B2 lineage, especially among isolates producing CTX-M enzymes, is of interest due to its worldwide success. The present prospective study investigated which factors among 66 studied were associated with those of ST131 *E. coli* clinical isolates that produce CTX-M enzymes. Among the various types of food products analysed, it was found, for the first time, that consumption of poultry meat at least twice a week is a factor inversely associated with isolation of a CTX-M-producing ST131 *E. coli* clinical isolate among the CTX-M-producing *E. coli* clinical isolates. In other words, it means that consumption of poultry meat was associated with isolation of CTX-M-producing *E. coli* that did not belong to ST131. This finding is of importance with regard to the debate on the potential food-borne source of *E. coli* ST131, notably those producing CTX-M enzymes [8], [16]. Poultry meat was suggested as a source of *E. coli* ST131 on the basis of two studies published in 2010 because *E. coli* ST131 has been isolated from poultry meat samples [7], [8]. The most recent studies conducted in the Netherlands and in Spain challenged this hypothesis as they failed to isolate CTX-M-producing *E coli* ST131 from chicken meat samples [3], [6]. Overall, the results of our study are in accordance with the fact that *E. coli* ST131 has not been identified among ESBL-producing *E. coli* isolated from retail chicken meat on the contrary to other lineage [3], [5], [6], [17]. Although, there are very few studies on the population structure of ESBL-producing *E. coli* isolates from poultry meat, it is noteworthy that, among the CTX-M-producing non ST131 *E. coli* clinical isolates, some dominant clonal groups (ST10, ST117 and ST354) are commonly identified from chicken meat [3], [8], [18]. Interestingly, ST167 and ST648, the two highest dominant clonal groups after ST10 among the CTX-M-producing non-ST131 *E. coli* clinical isolates had been identified among ESBL-producing *E. coli* isolates from Spanish poultry farms and from birds of prey from Germany and Mongolia [19], [20]. In summary, the dominant non-ST131 clonal groups in our population are clonal groups commonly identified in avian populations.

The only factor positively associated with isolation of CTX-M-producing *E. coli* ST131 from clinical samples was residence in LTCF before inclusion in the study. Rooney *et al*. showed that a high proportion of people living in such settings in England had digestive tract colonization with ESBL-producing *E. coli* ST131 [21]. Of note, the first identification of CTX-M-15-producing *E. coli* ST131 in France was achieved from patients in LTCFs [22], [23]. More recently, Banerjee et al. conducted a retrospective study in all healthcare settings in Olmsted County (Minnesota) and found that LTCF residence was a factor independently associated with *E. coli* ST131 [24]. Overall, three studies conducted in three different developed countries have found a link between LTCF residence and *E. coli* ST131. This might suggest that human cross-transmission is a key factor in the dissemination of CTX-M-producing *E. coli* ST131.

Although the proportion of hospital-acquired (isolation after 48 h of hospitalisation) CTX-M-producing ST131 and non-ST131 *E. coli* isolates was high and not significantly different (63.6% *vs* 56.2%: P = 0.2) and the patients infected by either *E. coli* ST131 or *E. coli* non-ST131 did not differ with regard to Mac Cabe score, we found that presence of invasive devices in the preceding six months and stay in ICU before study inclusion were inversely associated with isolation of CTX-M-producing *E. coli* ST131. It suggests that isolation of CTX-M-producing non-ST131 *E. coli* from clinical samples is more likely to be healthcare-related. Such results seem to be in contradiction with those obtained by Banerjee *et al*. [24]. Indeed, they found that *E. coli* ST131 is linked to healthcare and hospital acquisition. However, we noted that this link was identified by Banerjee *et al* in the univariate and not in the multivariate analysis that they carried out.

Finally, we were not able to link travel abroad, notably in Africa and India, to isolation of CTX-M-producing *E. coli* ST131 from clinical samples probably because of the lack of power regarding this association in our study [25].

In conclusion, this study provides more insight into the epidemiological features of ST131 and non-ST131 *E. coli* producing CTX-M enzymes. It shows, for the first time, that isolation of CTX-M-producing *E. coli* ST131 from clinical samples was not linked to consumption of specific foods and confirms that residence in long term care facilities is linked to these isolates. Further studies are required to know whether our results are also relevant for *E. coli* ST131 not producing CTX-M enzymes.
